# Head Accelerations during a 1-on-1 Rugby Tackling Drill Performed by Experienced Rugby Union Players

**DOI:** 10.3390/brainsci11111497

**Published:** 2021-11-12

**Authors:** Tahere Reha, Colm McNabb, Kevin Netto, Paul Davey, Andrew P. Lavender

**Affiliations:** 1 Curtin School of Allied Health, Curtin University, Perth, WA 6102, Australia; tahere.reha@graduate.curtin.edu.au (T.R.); colm.mcnabb@graduate.curtin.edu.au (C.M.); kevin.netto@curtin.edu.au (K.N.); p.davey@curtin.edu.au (P.D.); 2 Curtin enAble Institute, Curtin University, Perth, WA 6102, Australia; 3 Curtin School of Nursing, Curtin University, Perth, WA 6102, Australia; 4 School of Science, Psychology and Sport, Federation University Australia, Ballarat, VIC 3350, Australia

**Keywords:** rugby union, tackling, contrecoup effect, concussion, subconcussion, traumatic brain injury, neck injury

## Abstract

Rugby Union is a popular sport played by males and females worldwide, from junior to elite levels. The highly physical skill of tackling occurs every few seconds throughout a match and various injuries associated with tackling are relatively common. Of particular interest are head injuries that result in a concussion. Recently, repeated non-injurious head impacts in sport have attracted the attention of researchers interested in brain health. Therefore, this study assessed head movement during repeated rugby tackle drills among experienced Rugby Union players. Experienced male and female participants performed 15 1-on-1 tackles in a motion analysis laboratory to measure the head movements of the ball carrier and tackler during each tackle, using three-dimensional motion capture. The average peak acceleration of the head for ball carriers was 28.9 ± 24.08 g and 36.67 ± 28.91 g for the tacklers. This study found that the type of head impacts common while performing a tackle in Rugby Union are similar to those experienced by soccer players during heading, which has been found to alter brain function that lasts hours after the event. This has important implications for player health and suggests that mitigation strategies should be considered for Rugby Union.

## 1. Introduction

Tackling is a fundamental component of Rugby Union (henceforth termed “rugby”). These high-intensity physical contests occur when an attacking player in possession of the ball is brought to the ground by one or more defending players [1]. The tackle is the most common contact event in rugby, with a single player exposed to between 10–25 tackles per game [2,3]. Due to the frequency and physicality of these contact events, players are exposed to a relatively high risk of injury, with tackling responsible for a large majority of all injuries in rugby [1,4]. Of particular concern is the risk of head injury, which can occur during a tackle and, despite specific rules to minimise the risk of head injury during a tackle, this event still has the highest association with head injury, responsible for 84% of all head injuries in rugby [5].

In recent decades significant research has focused on acute injuries associated with tackling, particularly concussion [6]. In fact, a relationship between concussion and long-term cognitive, neurobehavioral and psychiatric problems and the development of neurodegenerative disease has now been established [7,8,9]. Chronic traumatic encephalopathy (CTE) is one such neurodegenerative disease, which has been linked to athletes involved in contact sports including rugby [10]. Interestingly, the development of CTE has also been found in athletes involved in contact sports who have no history of concussion [11,12]. In response to this finding, current brain injury research has focused on the investigation of acute changes in brain function following subconcussive head impacts (those impacts which do not result in the diagnosis of concussion) [11,12]. For instance, an investigation of soccer athletes without any history of concussion demonstrated changes in white matter integrity compared with swimmers, suggesting a cumulative effect on the brain leading to long-term impairments as a result of repeated minor head impacts—deliberate heading—common in soccer [11].

Specific rules have been introduced to minimise the risk of head injuries in rugby. However, impacts to other parts of the body during contact events can result in inertial loading of the head and may be capable of producing enough force to result in structural injury to the brain [6]. Stabilisation of the head may be an important factor for reducing players’ risk of sustaining head injuries [13]. Dynamic stabilisation of the head is largely a result of the ability of the cervical musculature to attenuate loads in response to perturbation [14,15,16]. The muscles involved in neck flexion are the sternocleidomastoid, scalenus, longus capitus and rectus capitus anterior, and the muscles that contract for neck extension are the sternocleidomastoid, spinalis cervicis and spinalis capitus. Properties such as muscle strength, muscle mass and the timing of muscle activation have all been shown to attenuate the force and resultant head movement [17]. Significant associations with greater isometric neck strength and decreased head velocities in response to impulsive loads from all directions have been reported [14,18]. Also, significant association with neck strength and decreases in the magnitude of head impacts during soccer heading have been reported [13].

The direct measurement of head kinematics on the field is difficult to conduct in rugby. An alternative approach is to replicate a tackling drill in a motion analysis laboratory where an accurate measure of independent head movement in relation to the thorax can be obtained [19]. Therefore, the primary aim of this study was to measure three-dimensional head kinematics relative to the thorax during a 1-on-1 tackling drill in rugby. The secondary aim was to investigate the relationship between head acceleration and neck strength, neck girth and experience. We hypothesised peak head acceleration would be negatively correlated with neck strength, girth and experience.

## 2. Materials and Methods

A cross sectional design with repeated measures was used for this study and 18 healthy adults were recruited to participate (16 males and 2 females). All participants were active rugby players, registered with a Rugby Union club in Western Australia at the time of the study. Participants were deemed suitable and were included if they met the following inclusion criteria: above 18 years of age, have playing experience of at least one season and be a currently registered player of a Rugby Union club at the level of Colts (under 20′s), Premier, Reserve Grade or higher. Any volunteer with a current musculoskeletal injury limiting their ability to engage in high intensity physical contact was excluded from the study. The study was approved by Institutional Human Ethics committee (HR231/2015) and participants gave informed consent prior to participating.

**Figure 1:**
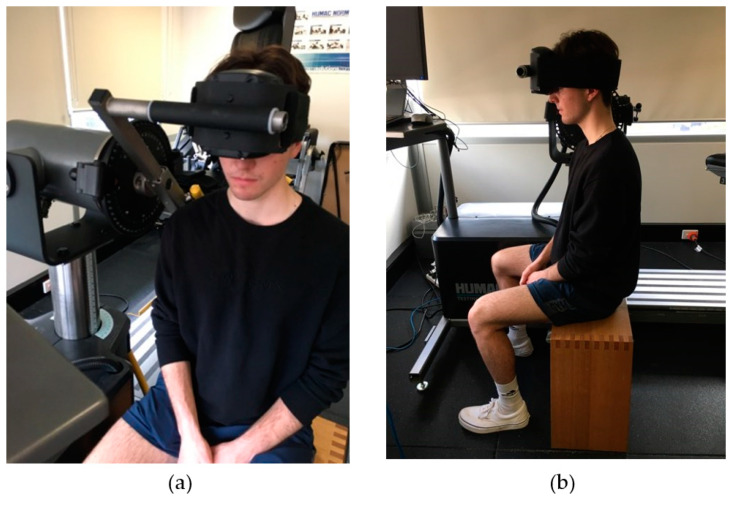
Set up of isokinetic dynamometer for isometric flexion and extension neck strength measurement front (**a**) and side (**b**) views.

A pre-testing session was conducted for all participants, during which, participants’ height and mass were measured using a stadiometer and electronic scale (Seca 284, Hamburg, Germany). Neck girth was measured at the level directly below the laryngeal prominence (Adam’s apple). Cervical muscle strength was measured with an isokinetic dynamometer (Humac Norm, Computer Sports Medicine, Inc., Stoughton, MA, USA). The head was positioned with the axis of rotation of the torque arm aligned with the participant’s seventh cervical vertebra (C7). Each participant was positioned with their forehead against the resistance pad and a Velcro strap wrapped around their head to secure it to the arm of the dynamometer to obtain peak isometric flexion torque (Figure 1). The participant was given one practice trial followed by three data trials with a 30-s rest between each attempt. The same procedure was then used for extension where the participant was asked to push their head back, in the opposite direction to obtain peak isometric extension torque. This method of strength testing has been shown to provide valid and reliable results [20,21]. The pre-testing session was conducted at least one week prior to the main testing; this was to allow for adequate rest of cervical musculature post-strength testing [20].

**Figure 2:**
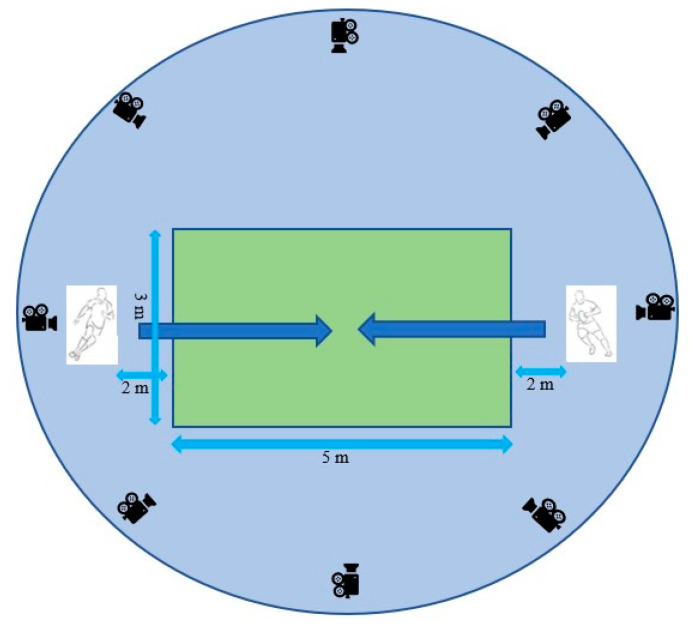
The laboratory layout for tackles. (Not all cameras shown). The ball carrier was instructed to try to get to the opposite side of the mats (shown in green) without going off the mats. The tackler was instructed to stop the ball carrier and bring him to the ground. Tackles were stopped if the ball carrier was not brought down within 5 s off tackle initiation.

Participants were allocated a partner based on sex, physical stature and competition level. The partners were then randomly allocated to be either the ball carrier or the tackler. The main testing session was completed in a motion analysis laboratory equipped with an 18-camera optical motion-capture system (Vicon VX, Oxford Metrics, Inc., Oxford, UK) (Figure 2). Prior to the arrival of the participants, the system was calibrated as per manufacturer specifications and all kinematic data was obtained at 250 Hz. Participants attended the main testing session with their allocated partner. Upon arrival, both participants were fitted with retro-reflective markers used for motion tracking. Two markers were placed posterior to the acromioclavicular joints, one on the C7 spinous process, two on the posterior superior iliac spine and one on the superior angle of the scapula for referencing. Another four markers were attached to a rugby helmet (Xact Headgear, Gilbert) worn by the participants to represent three-dimensional head movement. The rugby helmets used were identical in make and model and markers were placed in holes in the headgear in the same position for each participant. High-speed motion capture has been shown to be an effective way of quantifying accurate readings of head movement during a rugby tackle [6,19].

The laboratory was set up with padded mats in a 3 × 5 m area, with the participants positioned 9 m apart (each 2 m away from the mats) to replicate a 1-on-1 rugby tackling drill. The approach speed of the ball carrier was monitored using the motion capture software. The ball carrier was required to reach a minimum velocity of 4.5 ± 1 m/s which has been described as a typical approach speed for a ball carrier immediately prior to a tackle [4]. The instructions for the ball carrier were to reach the opposite side of the marked area as best they could without going outside the marked area. The tackler was instructed to stop the ball carrier from reaching the opposite side by tackling them. The players engaged in the task when a timer began and ceased when one of the following situations occurred: the ball carrier reached the opposite side; the tackler successfully tackled the ball carrier to the ground or the tackle came to a stand-still for a minimum of three seconds. At the end of the tackling scenario, both players were given a minute to recover and to return to the starting point before undergoing another tackle. This was repeated until 15 tackles were recorded.

Peak isometric neck flexion and extension torque recorded from the dynamometer, measured in newton-metres (Nm), and input into a spreadsheet. The data were normalised to body mass and a neck flexion-to-extension ratios were calculated. Raw kinematic data were converted to three-dimensional coordinate data using manufacturer-supplied software (Nexus; Oxford Metris, Inc., Oxford, UK). A customised LabVIEW program (2015 SPI, National Instruments Corp, Austin, TX, USA) was used to obtain head velocity vectors in three dimensions. The velocity vectors were then used to calculate the linear accelerations of the head relative to the thorax (HrT) and these accelerations were expressed in multiples of gravity (g).

Initially, a Shapiro–Wilk test determined the data was normally distributed. An independent t-test was conducted to determine any difference between the average HrT accelerations between tacklers and ball carriers. The mean of the peak flexion and extension torque generated (Nm) from the dynamometer was calculated over three trials. A correlational analysis between the peak isometric neck strength and the peak HrT accelerations was performed. A Pearson’s correlation was also conducted to examine the relationship between neck strength ratio (flexion:extension) and the peak HrT accelerations. Correlational analysis was conducted between HrT accelerations and neck girth and players’ experience, determined by the number of seasons played. All statistical analyses were conducted using SPSS (SPSS 17, Chicago, IL, USA.) with statistical significance set as *p* < 0.05.

## 3. Results

Participants were aged between 18 and 45 years (mean ± standard deviation; age 22.9 ± 6.3 years, height 179.5 ± 7.6 cm and mass 90.7 ± 13.2 kg) and ranged in playing experience from 1 to 26 years (mean ± standard deviation 12.5 ± 6.4 years). Participants’ neck girth was 40 ± 3 cm. Normalised peak isometric neck flexion torque was 0.3 ± 0.1 Nm/kg and normalised peak isometric neck extension torque was 0.6 ± 0.2 Nm/kg. Participants’ neck isometric flexion to extension torque ratio ranged from 0.33 to 0.93.

A total of 258 tackles of a possible 270 were successfully processed with 12 tackles discarded due to issues with displacement of markers during the tackling. The mean HrT for the tackling group was 36.67 ± 28.91 g while the mean HrT for the ball-carrier group was 28.9 ± 24.08 g. An independent Student’s T-test revealed no statistically significant difference in the HrT acceleration between tackler and ball carrier groups (*p* = 0.27).

Correlational analyses revealed a significant though moderate positive correlation between the linear acceleration of HrT and neck flexion torque (r = 0.48, *p* = 0.04), neck flexion-to-extension ratio (r = 0.5, *p* = 0.03) and neck girth (r = 0.54, *p* = 0.02). No significant correlation between HrT acceleration and neck-extension torque or players’ experience were recorded.

## 4. Discussion

This study investigated the head relative to thorax kinematics during a bout of 1-on-1 rugby tackling drills. Our results show that while there was no statistically significant difference in HrT between the groups, there was a tendency for slightly higher HrT in the tackling group compared with the ball carriers and peak neck-flexion torque, neck flexion-to-extension ratio and neck girth significantly correlated with head acceleration.

The HrT accelerations were similar to those reported in other laboratory and field-based investigations of head kinematics. In a laboratory-based study of soccer heading, Naunheim et al. [22] reported head accelerations of 15–20 g, while a field-based study of American Collegiate football tackling reported head accelerations of 32 ± 25 g (1–200 g) measured from a helmet-mounted accelerometer [23]. An investigation of in-field head acceleration in youth girls’ soccer reported data from player collisions being 22.3 ± 6.1 g (4.5–62.9 g) [24]. King et al. [25] reported 22.2 ± 16.2 g for head accelerations measured from an instrumented mouthguard from a single rugby team during a season of matches. In the present study, the approach velocity of the ball carrier was standardised. It is likely that tackles and collisions do not occur at the same velocity in the field and this could explain some of the variance when comparing results to field-based studies. Also, this study measured the acceleration of the head relative to the thorax while in-field measures from instrumented mouthguards or helmet-fitted accelerometers do not give this value, which leaves room for discrepancy between the data.

In an attempt to establish a threshold value of head acceleration for the diagnosis of concussion, Zhang et al. [26] used twenty-four reconstructed football field collisions to establish a probable threshold for causing mild traumatic brain injury. They concluded that linear accelerations of 66 g, 82 g and 106 g resulted in a 25%, 50% and 80% risk of concussion, respectively [26]. In the present study, there were 10 tackles that reached the magnitudes for 80% risk of a concussion. However, none of the participants reported nor demonstrated any of the symptoms of concussion. The relationship between sub-concussive head impacts in rugby and the development of long-term neurological impairments remains underexplored. However, data from a study that was undertaken in tandem with the present investigation assessed cortical inhibition, using transcranial magnetic stimulation, and showed deficits immediately after the bout of tackling among the tacklers but not the ball carriers nor a third group of controls who ran through the drill without performing the tackle component [27]. While both the tackler and ball carrier are exposed to sub-concussive impacts during simulated tackling, it appears that the tacklers in this drill are more prone to these minor changes in cortical function than the ball carriers even though the difference in HrT was not statistically different [27]. Other recent investigations in soccer ball heading and boxing sparring practice have found a similar acute disruption in cortical function, absent of any detectable changes, using standard sideline testing procedures that assess memory and balance [28,29]. For example, in a study by Di Virgilio et al. [29], boxers showed increased corticomotor inhibition, altered motor-unit recruitment strategies and reduced memory performance compared with controls one hour after the training bout with values returning to normal within 24 h.

In this study, the tacklers HrT acceleration was slightly higher than the ball carrier. Tierney and Simms [17] analysed 40 tackles from two randomly selected professional rugby matches and also showed the tackler to have higher head acceleration in both upper- and lower-body tackles compared with the ball carrier. The difference in head acceleration between the tackler and the ball carrier may be attributed to the anticipatory and bracing effects the ball carrier has before being tackled [6,17]. Another explanation for the difference may be due to the location of the impact on the body of the ball carrier and the tackler. It has been shown that the height of the tackle has a large impact on the resultant head acceleration of the ball carrier, as energy transmitted during an impact is attenuated along the kinetic chain [6]. As the location of impact can vary for the ball carrier (who, according to the current laws of rugby, may be contacted anywhere below the line of the shoulders), the resultant head acceleration will depend on the location of the tackle [6]. However, as the tackler must make contact with their upper body, often the shoulder, there is less potential for force dissipation and therefore, on average, the tackler experiences larger resultant head accelerations.

Our results showed a higher neck strength, a larger neck girth and a more symmetrical balance between neck flexion-to-extension strength had a moderate correlation with increased linear head acceleration. This is in contrast to the belief that greater neck strength and more symmetrical strength of neck flexors and extensors may reduce head acceleration during sporting impacts due to a greater ability to brace the head prior to impact [30]. Although this correlation is only moderate, our data indicates that higher neck strength may hinder a rugby player’s ability to attenuate acceleration to the head. There is data to suggest that neck strength has a minimal effect on reducing the acceleration of the head in the transverse plane and changes in neck strength have no effect on head velocity during head impacts [31,32]. Mihalik et al. [33] also showed isometric neck strength did not correlate with head velocity in hockey players. The timing of neck-muscle activation prior to an impact may be more important than neck muscle strength. A previous study suggested that activation of cervical musculature prior to an impending impact plays a significant role in reducing the acceleration of the head, as well as the risk of sustaining head injury [31]. Future studies may choose to investigate the role dynamic neck-strength and neck-muscle activation patterns have on attenuating head acceleration during a rugby tackle.

There were several limitations to the current study. Firstly, the tackles were conducted in a controlled, laboratory setting; therefore, they may not replicate the true nature of tackles that would occur in an on-field training drill or game situation. It can be anticipated that the linear accelerations of HrT would be higher than the present study, as athletes’ intensity during on-field tackles would be higher. This study provides good insight into what levels of acceleration these players are exposed to during training sessions. Further, our study only measured isometric neck strength; dynamic neck strength may have an impact on head kinematics during a tackle and, therefore, further investigation may be required to examine this relationship. Another consideration is that the majority of participants in this study were male. With an increasing number of female rugby players, it is important to have an understanding of the mechanics of females in the sport. Therefore, future studies should aim to investigate the differences between male and female athletes during a rugby tackle.

## 5. Conclusions

This study showed that tacklers may be exposed to higher HrT accelerations compared with ball carriers in a single bout of rugby tackling. Rugby players are exposed to a range of HrT accelerations during a 1-on-1 tackling drill that can place them at elevated risk of injurious and non-injurious head impacts. While this may have no, or very mild acute effects, the long-term consequences of such head accelerations remain unknown. Recent research has shown that there may be a cumulative effect of such impacts across a season or multiple seasons [34]. Additionally, while it is important to keep in mind that data obtained through animal studies do not translate directly to humans, the outcomes from such studies are useful to inform us about potential mechanisms regarding the long-term effects of multiple head impacts repeated over long periods [35,36]. Coaches and managers may choose to limit the volume and intensity of tackling drills to lower the risk of head injury among rugby players. This study also found no effect of neck strength on head movement during rugby tackling drills, suggesting that greater neck strength may not play a critical role in attenuating accelerations to the head during a tackle.

## Data Availability

The data presented in this study are available on request from the corresponding author. The data are not publicly available due to the inclusion of personal information of participants.
